# Sleep Problems and 6-Sulfatoxymelatonin as Possible Predictors of Symptom Severity, Adaptive and Maladaptive Behavior in Children with Autism Spectrum Disorder

**DOI:** 10.3390/ijerph19137594

**Published:** 2022-06-21

**Authors:** Kristina Bartakovicova, Petra Kemenyova, Ivan Belica, Zofia Janik Szapuova, Katarina Stebelova, Iveta Waczulikova, Daniela Ostatnikova, Katarina Babinska

**Affiliations:** 1 Academic Research Centre for Autism, Institute of Physiology, Faculty of Medicine in Bratislava, Comenius University, 813 72 Bratislava, Slovakia; kristina.bartakovicova@fmed.uniba.sk (K.B.); petra.kemenyova@fmed.uniba.sk (P.K.); ivan.belica@fmed.uniba.sk (I.B.); szapuova.z@gmail.com (Z.J.S.); daniela.ostatnikova@fmed.uniba.sk (D.O.); 2 Research Institute for Child Psychology and Pathopsychology, 831 05 Bratislava, Slovakia; 3 Department of Animal Physiology and Ethology, Faculty of Natural Sciences, Comenius University, 842 15 Bratislava, Slovakia; katarina.stebelova@uniba.sk; 4 Department of Nuclear Physics and Biophysics, Faculty of Mathematics, Physics and Informatics, Comenius University, 842 48 Bratislava, Slovakia; waczulikova1@uniba.sk

**Keywords:** autism spectrum disorder, children, core symptoms, sleep problems, melatonin, adaptive functioning

## Abstract

In children with autism spectrum disorder (ASD), sleep disturbances are a frequent comorbidity with an adverse effect on their behavior and functioning. It was suggested that melatonin deficit is at least partly responsible for the sleep problems. The study aimed to investigate, in a sample of 56 children with ASD aged 2.8–13.3 years, if the sleep problems and melatonin secretion can serve as predictors of adaptive functioning and severity of the ASD core symptoms. We demonstrated that, after adjustment for age, the Sleep score assessed by the Children’s Sleep Habits Questionnaire predicts the Adaptive behavior composite score only in children younger than 6 years, and the preferred predictive model is for the domain Socialization. The age-adjusted Sleep score predicted Externalizing and Internalizing maladaptive behavior, with a near-zero contribution of age to the relationship between the Internalizing maladaptive behavior and Sleep score. After adjustment for age, the reduced night-time melatonin secretion predicted a higher severity of ASD symptoms in the domain Social affect and the Calibrated Severity Score, but not the sleep problems. Our results emphasize the importance of assessing sleep problems as a modifiable predictor of behavior in children with ASD and support the hypothesis about the role of melatonin in pathophysiology of ASD.

## 1. Introduction

Autism spectrum disorder (ASD) is a group of neurodevelopmental disorders with multiple manifestations, characterized by impairments in reciprocal social interaction and communication skills, as well as by the presence of restricted and repetitive behaviors [1]. Children with ASD often display deficits in adaptive functioning that is defined as the collection of conceptual, social, and practical skills that are learned and are performed in everyday life [1]. In general, the subjects with ASD have a score in adaptive skills of one to two standard deviations below their peers of the same age [2]. A large body of evidence indicates that children with ASD suffer from many medical comorbidities [3]. Sleep disturbances are one of the most common comorbid conditions, with prevalence rates ranging from 50 to 80% [4,5].

Even in typical development, sleep disturbances are associated with emotional and behavioral problems, including anxiety, depression, or aggression [6], but the daytime dysfunction may be especially challenging for children with ASD because of their unique behavioral characteristics [7]. It has been shown that insufficient sleep is correlated with the severity of ASD core symptoms [8,9] and higher rates of repetitive behaviors, as well as with social skill deficits and communication impairments [10,11,12]. Only a few studies have investigated the relationships between sleep problems and adaptive performance in individuals with ASD [5,7,8,13,14,15]. They have shown associations of sleep impairments with challenging behaviors, such as overactivity, non-compliance, irritability, or aggression. Hollway et al. [8] observed a strong relationship between sleep problems and internalizing (e.g., anxiety, depression) and externalizing (e.g., aggression) maladaptive behaviors. In addition, Sikora et al. [5], reported that children with ASD and moderate–severe sleep disturbances had poorer adaptive skill functioning and higher daytime externalizing behavior than those individuals without sleep disturbances. In a study evaluating the adaptive behavior of young children with ASD, Krakowiak et al. [13] reported no association between adaptive delays and sleep problems, while some other studies revealed that children with a shorter sleep duration had more difficulties with completing daily living activities, they had lower overall intelligence and verbal skills, as well as poorer motor and social skills [9,15].

Melatonin as a major regulator of the circadian rhythm plays an important role in sleep disturbances [16]. It has been shown that a proportion of children with ASD display abnormalities in melatonin production [17]. In a study by Leu et al. [18], the lower overnight levels of 6-sulfatoxymelatonin (aMT6s), the main metabolite of melatonin excreted by urine, were negatively correlated with daytime sleepiness. In addition, dysfunction in the sleep initiation and maintenance correlated with night-time urinary aMT6s excretion [19]. It was also observed that low diurnal as well as nocturnal aMT6s levels in the urine of children with ASD correlated with more severe impairments in verbal communication, social skills, and stereotyped behavior [20,21].

The aim of our study was to analyze if the sleep problems in children with ASD, as well as the concentration of 6-sulfatoxymelatonin in their spot urine samples as a potential objective marker associated with sleep disturbances, can serve as predictors of the adaptive functioning and severity of the ASD core symptoms.

## 2. Materials and Methods

### 2.1. Sample

**Figure 1:**
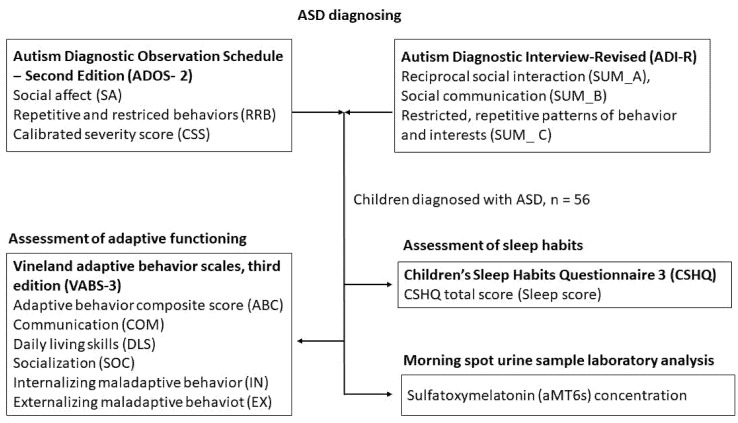
Diagram showing the overview of examinations and variables.

The study involved 56 participants (50 boys and 6 girls) aged 2.8–13.3 years who were newly diagnosed with ASD at the Academic Research Centre for Autism (ARCA) at the Faculty of Medicine, Comenius University, Bratislava, Slovak Republic. The exclusion criteria included severe psychiatric or neurological comorbidities, associated genetic disorders, and the use of melatonin supplements. An overview of the examinations of the study participants and the variables included in the statistical analysis is illustrated in Figure 1.

### 2.2. Diagnostic Evaluation of ASD

The ASD diagnosis, according to the International Classification of Diseases (ICD-10), was confirmed by trained examiners using the Autism Diagnostic Observation Schedule, Second Edition (ADOS-2) and the Autism Diagnostic Interview-Revised (ADI-R).

The ADOS-2 is a standardized, semi-structured observational method to evaluate communication, social interaction, play and repetitive behavior, and restricted interests. It consists of five modules according to the level of expressive language and the age of the examined person. In our study, the participants met the criteria to be examined with modules 1, 2, or 3. The total score includes two domains: Social affect (SA) and Restricted and repetitive behavior (RRB) [22]. The calibrated severity score (CSS) was calculated allowing for the comparison of the severity of ASD symptoms across different modules [23]. Higher scores indicate a higher severity of symptoms.

The ADI-R is a semi-structured comprehensive interview with the parents or primary caregivers that provides a thorough assessment of the development and current condition of a child. The ADI-R focuses on qualitative abnormalities in three functional domains: Reciprocal social interaction (SUM_A); Social communication (SUM_B); and Restricted, repetitive patterns of behavior and interests (SUM_C) [24]. Due to the possible effect of age on the raw score, the final score in each domain was transformed to a ratio between the child’s raw score and the maximum possible score that could be achieved, and then expressed in percent (%). Higher values indicate a higher severity of symptoms.

The ASD diagnosis was made based on clinical evaluation; and all of the study participants had to meet the criteria for ASD in both of the diagnostic tools.

### 2.3. Assessment of Adaptive Functioning

For the assessment of adaptive functioning, the Vineland adaptive behavior scales, third edition (VABS-3) were used [25]. The VABS-3 is a semi-structured interview with the primary caregivers of children focused on adaptive behavior. Sparrow et al. [25] define adaptive behavior as fulfilling activities necessary for functioning in everyday life, or, in other words, an individual’s ability to meet the standards of social responsibilities and independence. The VABS-3 includes three main domains: Communication (COM, including receptive, expressive, written communication); Daily living skills (DLS, including personal, domestic, community skills); and Socialization (SOC, including interpersonal relationships, play and leisure time, coping skills). In addition to these individual scores, the output score includes an Adaptive behavior composite score (ABC) that is a general score of adaptive behavior. The VABS-3 standard scores have a mean of 100 and standard deviation of 15. Scores ranging from 85 to 115 are considered to be within the normal range, while scores lower than 70 are considered to be within the impaired range of ability. Thus, higher scores represent better adaptive functioning. The other part of the VABS-3 are two scales of maladaptive behavior, that include internalizing and externalizing challenging behavior. Internalizing maladaptive behavior includes impairments, such as different disruptive emotional affects, sleep, or feeding problems, while externalizing maladaptive behaviors include mainly different forms of verbal or physical aggressive attacks against people or objects. The mean score for maladaptive behavior is 15, and the standard deviation is three. Higher scores suggest a higher severity of maladaptive behavior. All of the raw scores were transformed into standard scores, which allows a comparison of the scores across different age groups.

### 2.4. Assessment of Sleep Habits

The sleep habits of the study participants were measured by the Children’s Sleep Habits Questionnaire 3 (CSHQ), that is a 45-items validated retrospective parent questionnaire. It includes eight subscales (Bedtime Resistance, Sleep Onset Delay, Sleep Duration, Sleep Anxiety, Night Waking, Parasomnias, Sleep-Disordered Breathing, and Daytime Sleepiness). The parents used a three-point scale for the answers: 3 = usually (if the sleep problem occurred five–seven times per week); 2 = sometimes (two–four times per week); 1 = never/rarely (0–1 time per week). The CSHQ total score (Sleep score) is calculated as a sum of the scores in the subscales. A CSHQ total score ≥ 41 is suggested to be the clinical cut off for a possible sleep dysfunction. Higher scores indicate more severe sleep problems. The CSHQ is a widely used sleep assessment method for children with ASD [26].

### 2.5. Urine Collection

The parents collected the participants’ first morning urine at home, and delivered it immediately to the laboratory. The urine samples were stored at −18 °C until analysis. The levels of 6-sulfatoxymelatonin (aMT6s) in the urine were determined by a melatonin sulfate ELISA kit (DRG Instruments, Marburg, Germany). The assay was performed according to the manufacturer’s instructions. The kit’s control levels were found to be within the acceptable ranges, as stated on the quality control certificate. All of the samples were measured within four assays. The mean intra-assay coefficient was 6.12%. The mean inter-assay coefficient was 10.65% and 19.9% for the Control 1 and Control 2 samples, respectively. The levels of the aMT6s were normalized on creatinine in each urine sample (aMT6s/creatinine) to control for variations in the urinary flow rate [27]. The creatinine level in the urine was determined using an enzymatic method for quantitative in vitro determination of creatinine in human serum, plasma, and urine (Erba Lachema, Brno, Czech Republic). The kit was used according to the manufacturer´s instructions. The results are presented as ng of aMT6s in mg of creatinine in each urine sample.

### 2.6. Statistical Analysis

The patients’ data were summarized using descriptive statistics. The continuous variables were first checked for normality, using graphical methods and the Shapiro–Wilk test. The skewed variables (e.g., age and creatinine-normalized urinary aMT6s concentrations) were transformed, using the natural logarithm (ln) function to improve the normality and homogeneity of variance, both requirements for the subsequent linear regression analysis. As a part of a bivariate descriptive statistic, correlation analysis was performed.

First, the univariate and bivariate analyses were performed to capture the associations between the variables. Univariate between-group differences in continuous variables were tested, using the Mann–Whitney test or nonparametric analysis of variance. An association in the categorical variables grouped in two-way contingency tables was analyzed, using chi-square tests. In the case of numerical calculability, the exact tests were applied. Following an analysis of the simple relationships, multivariable modeling was carried out.

Based on the univariate analysis results, multivariable regression was performed to identify the unique (independent) contributions of each relevant predictor (after adjusting for potential confounders) on the outcome—the scores from the autism diagnostic tools ADOS-2, ADI-R, and VABS-3 related to behavioral issues.

We ran several models based on the nature of the outcome—multiple linear regression and multiple logistic regression. The continuous predictors (covariates), such as creatinine-normalized aMT6s level, were centered and normalized to facilitate the interpretation of the estimates. To explore whether the relationships between the predictor and outcome variables were linear or non-linear, we included Age first, as a continuous variable, and secondly, as a categorical variable. We categorized Age into three categories: 2–3, 4–5, and 6+ years.

For the data processing and analysis, the software Microsoft Office Excel 2016 (Microsoft Corporation, Redmond, Washington, DC, USA), Statistica 13.1 (TIBCO Software Inc., Palo Alto, CA, USA) and StatsDirect^®^ 3.3.5 were used (StatsDirect Ltd., Cheshire, UK). All of the tests were two-tailed and conducted at a significance level α  =  0.05.

## 3. Results

Table 1 presents the main characteristics of the sample. They include the behavioral characteristics, represented by the score in three functional domains assessed by the ADI-R: Reciprocal social interaction (SUM_A); Social communication (SUM_B); and Restricted, repetitive patterns of behavior and interests (SUM_C), as well as the scores based on the ADOS-2 diagnostic tool: Social affect (SA); and Restricted and repetitive behavior (RRB), and the Calibrated Severity Score (CSS). The score in the VABS-3 includes three main domains: Communication (COM); Daily living skills (DLS); and Socialization (SOC), as well as the scores for internalizing (IN) and externalizing (EX) maladaptive behavior. The sleep characteristics of the sample are represented by the CSHQ Sleep score, based on the Children’s Sleep Habits Questionnaire (CSHQ).

### 3.1. Explorative Correlation Analysis

**Figure 2:**
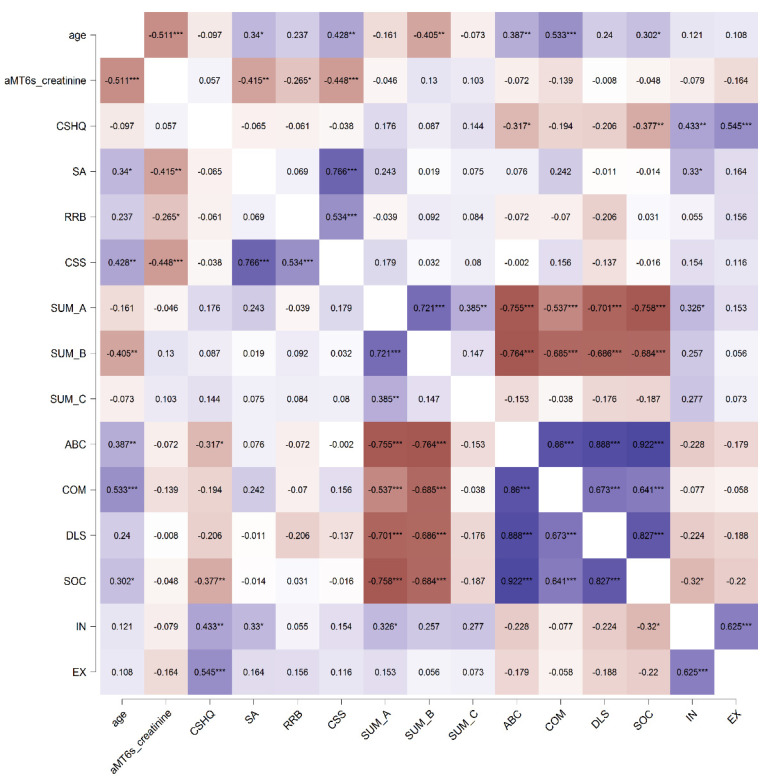
Heatmap based on simple pairwise Spearman correlation coefficients (ρ) in the ASD group. Violet color indicates positive correlations, brown color indicates negative correlations, a higher color intensity depicts stronger correlations. Statistical significance is denoted by asterisks as follows: * *p* < 0.05, ** *p* < 0.01, *** *p* < 0.001. ABC—Adaptive behavior composite score; aMT6s/creatinine—Creatinine-normalized urinary aMT6s; COM—Communication; CSHQ—Children’s Sleep Habits Questionnaire—Sleep score; CSS—Calibrated Severity Score (ADOS-2); DLS—Daily living skills; EX—Externalizing maladaptive behavior; IN—Internalizing maladaptive behavior; RRB—Restricted and repetitive behavior (ADOS-2); SA—Social affect (ADOS-2); SOC—Socialization; SUM_A—Qualitative abnormalities in reciprocal social interaction (ADI-R); SUM_B—Qualitative abnormalities in communication (ADI-R); SUM_C—Restricted, repetitive patterns of behavior and interests (ADI-R).

The study aimed to analyze, in a sample of children with ASD, if the sleep problems and the concentration of 6-sulfatoxymelatonin (aMT6s) in their spot urine samples can serve as predictors of adaptive functioning and the severity of the core symptoms. Firstly, an explorative correlation analysis between the variables was performed. Figure 2 shows a heatmap displaying the direction and strength of the correlations between the analyzed variables, based on Spearman’s correlation coefficients (denoted by the Greek letter rho/ρ). The creatinine-normalized urinary aMT6s (aMT6s/creatinine) significantly negatively correlated with all of the domains of ASD core symptom severity, according to ADOS-2: SA—Social affect (ρ = −0.415; *p* = 0.001); RRB—Restricted and repetitive behavior (ρ = −0.265; *p* = 0.048); and CSS—Calibrated Severity Score (ρ = −0.448; *p* = 0.001). The correlation of the aMT6s/creatinine with the ADI-R functional domains, adaptive behavior, and maladaptive behavior was not significant. Similarly, no significant correlation was found between the CSHQ Sleep score and the urinary level of normalized aMT6s. The Sleep score significantly negatively correlated with the VABS-3 Composite adaptive behavior (ρ = −0.317; *p* = 0.02), and its Socialization domain (ρ = −0.377; *p* = 0.005), and positively correlated with both Internalizing (ρ = 0.433; *p* = 0.002) and Externalizing (ρ = −0.545; *p* = 0.000) maladaptive behavior. However, no significant correlation was found between the Sleep score and ASD core symptoms severity determined by ADOS-2 and ADI-R assessment tools.

The concentrations of aMT6s/creatinine, as well as the behavioral markers, such as Social affect (SA), Calibrated Severity Score (CSS), qualitative abnormalities in communication (SUM_B), Adaptive behavior composite score (ABC), Communication (COM), and Socialization (SOC), displayed significant correlations with age (Figure 2). This raised a question, if the observed significant correlations are not primarily a result of the age differences of the participants, and whether they remain significant after adjustment for age. Therefore, as a next step, a multivariable analysis was performed. Since the variable Age was not normally distributed, we used three age categories (2–3, 4–5, 6+ years) in the analyses, with the lowest category as a reference.

### 3.2. aMT6s/Creatinine as a Predictor of ASD Symptom Severity

We have shown that the morning levels of aMT6s/creatinine (on a log scale) are associated with Social affect, determined with the ADOS-2 diagnostic tool. After adjustment for the age categories, the partial correlation coefficient yielded r = −0.294 (*p* = 0.031). The resulting predictive model was significant (*p* = 0.012) with multiple correlation coefficient R = 0.434. The same pattern of relationship was found for the morning aMT6s/creatinine levels and the Calibrated Severity Score (r = −0.329; *p* = 0.015), with a significant multiple correlation coefficient (R = 0.512; *p* = 0.001). However, we found only a weak negative correlation between the aMT6s/creatinine and Restricted and repetitive behavior (partial correlation coefficient r = −0.155; *p* = 0.264), and the model was not significant (*p* = 0.385; multiple correlation coefficient R = 0.237).

### 3.3. CSHQ Sleep Score as a Predictor of Adaptive Functioning

The CSHQ Sleep score showed a weak negative correlation with the Adaptive behavior composite score (ABC) (r = −0.119; *p* = 0.402). However, in comparison with previous models, we found a strong independent contribution of the highest age to the ABC (r = 0.509; *p* < 0.001) only. The middle age and the reference (youngest) category were not associated with the ABC score (r = 0.083; *p* = 0.561). The whole predictive model was statistically significant (*p* < 0.001) with the multiple correlation coefficient R = 0.557, implying that the Sleep score as a predictor might contribute and improve the performance of more complex models for the prediction of the degree of adaptive behavior. This might be true especially for the younger patients (up to six years), where the deficits of adaptive functioning due to more severe sleep problems cannot be outweighed by the gain in adaptive skills coming with age and increasing maturity. Since the variable Age seemed to produce an interaction effect, which means that it moderated the relation between the Adaptive behavior composite score (ABC) and the Sleep score, we performed a stratified analysis, according to the age categories. As expected, we found a significant inverse relationship between those two variables only in the subjects aged less than 6 years (r = −0.329; *p* = 0.041). In children aged 6 years or more, the relationship between the Adaptive behavior composite score (ABC) and the Sleep score was not significant (r = 0.181; *p* = 0.519).

A closer inspection of the data revealed that the main contribution to the observed weak negative relationship between the ABC score and the Sleep score came from the independent effect of the Sleep score on the domain, Socialization (SOC). After adjustment for the age categories, the partial correlation coefficient between the Sleep score and SOC yielded r = −0.298 (*p* = 0.032). The resulting predictive model was significant (*p* = 0.007) with a multiple correlation coefficient R = 0.461. The models for prediction of the other two domains, Communication (COM) and Daily living skills (DLS), using an age-adjusted Sleep score did not yield a significant independent contribution for that predictor (r = −0.012, *p* = 0.932 and r = −0.030, *p* = 0.831, respectively). The main contributing variable was the older age (the highest age category) with r = 0.588; *p* < 0.001, and r = 0.342, *p* = 0.013, respectively. The multiple correlation coefficient was only slightly higher (R = 0.609 and 0.408, respectively), thus confirming that the Sleep score did not bring information above that obtained from the relationship between Age and COM. On the contrary, these weak relationships lowered the predictive performance of the age-adjusted model for predicting an Adaptive behavior composite score (ABC) from the Sleep score. The preferred model is, thus, that for SOC, using the age-adjusted Sleep score as a predictor.

### 3.4. CSHQ Sleep Score as a Predictor of Maladaptive Functioning

We found a moderate, but significant, unique correlation between Internalizing maladaptive behavior and the age-adjusted Sleep score, as estimated with the partial correlation coefficient (r = 0.441; *p* = 0.002). The predictive model was significant, with the multiple correlation coefficient of 0.443, implying a near-zero contribution of Age on the observed relationship between Internalizing maladaptive behavior and the Sleep score (*p* = 0.021). We also found a moderate to strong correlation between Externalizing maladaptive behavior and the age-adjusted Sleep score. The partial correlation coefficient was r = 0.512 (*p* < 0.001) and the multiple correlation coefficient equaled R = 0.517 (*p* = 0.003).

To conclude this part, both of the predictive models worked sufficiently well on both scales, numerical as scores, and dichotomous, when the patients were classified as having or not having maladaptive behavior in the respective domains. We have found that the model for predicting Externalizing maladaptive behavior from the Sleep score and Age outperformed in classification accuracy that for predicting Internalizing maladaptive behavior.

## 4. Discussion

Adaptive behavior represents the individual’s ability to translate cognitive potential into real-life skills [25]. These skills include socialization, communication, and daily living skills [28]. In neurotypical children, adaptive behavior improves with age and, as a child grows older, adaptive behavior becomes more complex, implicating that age is a significant factor with an effect on the adaptive behavior. Children at high risk for ASD have been reported to perform lower in adaptive behavior at 20 months, 36 months, as well as in the middle of the childhood [29,30,31]. Adaptive behavior can be learned, and better adaptive skills increase the probability of the social inclusion of the individual and the improvement of his/her quality of life [32]. Therefore, it is important to determine the factors that may influence the adaptive behavior of an individual with ASD in early childhood [33]. In subjects with ASD, adaptive behavior has been shown to be more strongly correlated with optimum outcomes in adulthood than cognitive functioning [34]. Thus, the adaptive behavior is an important component in a comprehensive evaluation of ASD and a determinant of prognosis [35,36,37].

The existing evidence shows that sleep characteristics are associated with the behavior of children with ASD [5,14,16]. The aim of our study was to analyze if sleep problems can serve as a predictor of adaptive functioning. Our explorative data analysis revealed that the presence of sleep problems is associated with impairments in adaptive performance, namely in the VABS-3 Adaptive behavior composite score (ABC) and its domain Socialization (SOC). At the same time, both the ABC and SOC significantly correlated with age, indicating that the observed correlations might primarily result from the age differences of the participants. Therefore, a multivariable analysis was performed to test the predictive value of the age-adjusted Sleep score. It showed that the Sleep score is a significant predictor of the ABC only in children aged up to six years. In other words, that children with ASD in this age category, who presented with sleep problems, had poorer adaptive functioning and that their deficits in adaptive functioning due to more severe sleep problems cannot be outweighed by the gain in adaptive skills coming with age and increasing maturity. Our results indicate that there is a need to consider the age of the children with ASD when predicting adaptive functioning based on the sleep problems. Of all of the domains of the ABC, the main contribution resulted from the independent effect of the Sleep score on the domain Socialization (SOC); whereas the domains Communication (COM) and Daily living skills (DLS) reduced the predictive power for predicting the ABC from the Sleep score. Thus, concerning the use of the Sleep score as a predictor of adaptive functioning, the preferred model is that for the SOC domain using the age-adjusted Sleep score as a predictor.

Taylor et al. [15], observed that children who, on average, slept less per night had lower overall adaptive functioning, daily living skills, socialization, and motor skills. Yang et al. [38] also reported that children with sleep disturbances had more severe autistic traits, poorer overall adaptive behavior, daily living skills, social communication, and cognitive abilities than individuals without sleep problems. In a study of Sikora et al. [5], the participants were divided based on their CSHQ scores into groups of good and poor sleepers. The poor sleepers were further classified into two categories—the mild sleep problem group and the moderate to severe sleep problem group. They found that sleep disorders were associated with poorer communication and socialization skills in all of the participants, but daily living skills significantly correlated only with the moderate to severe sleep problem group. On the other hand, a study of Krakowiak et al. did not report a significant correlation between the adaptive behavior and severity of sleep problems in the ASD group [13].

Cohen et al. [39,40] contributed an interesting insight to the domain of sleep problems in ASD. They investigated whether challenging behaviors can be predicted from prior sleep patterns [40]. Based on the analysis of 20,000 nightly sleep observations and the occurrence of various types of behaviors during the day, they constructed a predictive real-time model between prior sleep and subsequent daytime behavior. The prediction accuracy increased with the number of previous nights on which the prediction was based, up to approximately 8 days. In another study [39], a cluster analysis was used to find different sleep patterns and relate them to an independent assessment of adaptive behavior in 106 children with ASD. The results suggested that cluster analysis may be useful to create clinically important subgroups of ASD individuals, on the basis of their sleep–wake behavior. The authors found two clearly different sleep phenotypes—unstable sleepers and stable sleepers. The unstable sleepers displayed lower IQ scores, as well as lower overall adaptive functioning scores (in the domains of communication, socialization, daily living skills, and the adaptive behavior composite score) when compared with the stable sleepers.

The VABS-3 also includes an evaluation of maladaptive behavior. In the multivariable analysis, we found a moderate, but significant, positive correlation between Internalizing maladaptive behavior and the age-adjusted Sleep score, where the contribution of Age was close to zero. This indicates that the sleep difficulties predict the probability of presentation with internalizing maladaptive behavior in children with ASD, irrespective of their age. Similarly, we demonstrated that Externalizing maladaptive behavior correlates with the age-adjusted Sleep score, i.e., the presence of sleep problems predicts the presentation of the externalizing maladaptive behavior in ASD, moreover, the model for predicting Externalizing maladaptive behavior from the age-adjusted Sleep score even outperformed the model for predicting Internalizing maladaptive behavior in classification accuracy.

Our results correspond with other studies, which have demonstrated that those children with ASD displaying sleep problems had greater internalizing (anxiety, inattention, emotional reactivity) and externalizing (aggression, hyperactivity, poor impulse control) behavior problems than the children without sleep problems [5,12,41,42]. For example, Malhi et al. [42] reported that the frequency of night awakenings and sleep disturbances were significant predictors of daytime challenging behaviors. Park et al. [10], in their study, also noted a significant correlation between sleep disturbances and withdrawal problems, internalizing problems, and aggressive behavior. Lambert et al. [43], using polysomnography as an objective method for sleep evaluation, reported notable findings. The children with ASD, who showed less slow-wave sleep, had more internalizing behavior. In addition, the proportion of slow-wave sleep negatively correlated with the social affect domain measured with ADOS. Adams et al. [44] reported that the presence of sleep problems was associated with greater scores for challenging behavior in individuals with ASD.

In summary, our findings support the evidence that poor sleep may exacerbate the challenging behavior. Maladaptive behavior in children with ASD is disruptive to the individuals and their caregivers, and it decreases the quality of life in various ways. The treatment of sleep problems may be effective in initiating changes in behavior. The studies have shown that interventions, such as parent-based education or pharmacological treatments, improve sleep disorders and can lead to a reduction in the severity of challenging behaviors in ASD [16,45,46]. In addition, according to the bidirectional theory, the effects of sleep disorders and behavioral problems are reciprocal [44,47,48,49,50]. A child who has a sleep problem is likely to be more irritable and hyperactive, but, conversely, an irritable or hyperactive child may have difficulties with sleep [51]. Reducing the challenging behavior in children, such as decreasing anxiety or hyperactivity, results in better sleep. Still, additional research is needed to clarify the bidirectional relationship.

The questionnaire methods for the evaluation of sleeping habits and assessment of sleep problems of children are commonly used, however, they may be biased by personal factors [52]. Melatonin is a physiological regulator of the circadian rhythm, and it plays a role in the pathophysiology of sleep disturbances [16], moreover, evidence indicates that a proportion of children with ASD exhibit abnormalities in melatonin production [17]. Rossignol and Frye, in their meta-analysis, report that the levels of melatonin or its derivatives are often below average in ASD [53]. Some of the studies have shown abnormalities in daytime melatonin levels, whilst in other studies lower nighttime melatonin secretion was observed or an abnormal circadian rhythm of secretion [53]. In a recent study, subgroups of ASD children were detected, some of them displaying a nighttime increase in melatonin, while others did not present the day-to-night difference in melatonin levels [19]. It has been shown that the peak nocturnal plasma melatonin was significantly related to urinary melatonin or aMT6s levels. Thus, the urinary measure shows a good sensitivity and specificity in identifying individual differences in the nocturnal plasma melatonin levels [54].

Our study investigated the urinary levels of normalized aMT6s, a waste product reflecting melatonin secretion. In the present study, no significant correlation between morning aMT6s/creatinine and sleep difficulties was observed. In our previous study, we investigated the melatonin secretion patterns in children with ASD and neurotypical individuals. Our results suggested lower nocturnal levels and a smaller circadian variation in melatonin concentration in ASD. However, even in this study, we have not found a significant correlation between melatonin levels and sleep problems [55]. In the study by Leu et al. [18], nocturnal urinary aMT6s levels were negatively correlated with a daytime sleepiness subscale, but no significant relationship was found between the Sleep score (CSHQ) and any other CSHQ subscale. Some of the other studies investigating this relationship in ASD individuals reported no correlations, but there are also studies that observed a negative correlation, indicating that lower melatonin levels are associated with more severe sleep problems [19,49].

We have demonstrated, however, that, after adjustment for age categories, the normalized aMT6s levels display a significant negative correlation between the ADOS-2 categories Calibrated Severity Score and Social Affect, resulting in significant predictive models. Thus, the lower morning levels of aMT6s predict a higher severity of ASD in the respective domains. Similar to our study, Tordjman et al. [20] found correlations of aMT6s with impairments in total and verbal communication, as well as in the play category based on the ADOS diagnostic tool. Regarding adaptive functioning as another behavioral aspect of ASD, our results do not support the role of the normalized aMT6s as a potential objective biomarker and predictor of maladaptive behavior, or adaptive functioning in general.

Melatonin is a neurohormone produced primarily in the pineal gland. The circadian rhythm of melatonin is characterized by low levels during the day in the conditions of light exposure, with a marked increase in the night, triggered by the darkness [56]. The major role of melatonin is to regulate the circadian rhythm, in particular the sleep–wake and core body temperature cycle; however, it is involved in a variety of other considerable functions, such as anti-inflammatory, antioxidant, immunomodulatory, and also neuroprotective effects [57,58]. Abnormal melatonin secretion may participate in the pathophysiology of ASD and its behavioral presentations, not only by exerting an effect on the sleep–wake cycle, but also via the additional mechanisms mentioned above. It should be noted that dysregulation of melatonin can also be related to ASD comorbid conditions, such as altered gastrointestinal motility, anxiety disorders, as well as sensory processing dysfunction. Extensive research indicates that melatonin by antioxidant function prevents cells from oxidative damage [59]. Indeed, several studies described increased oxidative stress in individuals with ASD [60,61]. Previous studies investigating the correlations of melatonin with ASD core symptoms revealed a negative correlation between nocturnal aMT6s excretion and verbal communication, imitative social play, and the repetitive use of objects [20,21]. Jonsson et al. [62] observed an association between the ASMT (Acetylserotonin O-methyltransferase), also known as HIOMT (Hydroxyindole O-methyltransferase), gene and social interaction impairments, but not with the ASD traits related to communication impairments and restricted, repetitive behavior. ASMT is the last enzyme in the synthesis of melatonin, and genetic variants in ASMT have been described in ASD. Pagan et al. [63], on the basis of previously reported alterations of melatonin and hyperserotonemia in individuals with ASD, aimed at the serotonin/N-acetylserotonin/melatonin pathway in patients with ASD, first-degree relatives, and sex and age-matched controls. They confirmed hyperserotonemia, increased platelet NAS levels, and a deficit in melatonin production in the ASD group. Intermediate levels were reported in the group of relatives, compared to the ASD and control groups. Other research groups also focused on the relatives of ASD patients. In addition, a recent study of Braam et al. [64] found significant lower melatonin levels in the mothers of ASD children compared to controls. It suggests the hypothesis that abnormal melatonin secretion can play a role in the pathomechanisms of ASD and can play a possible role as a biomarker in ASD. However, a causal link between ASD and melatonin has not yet been established.

Consistently with some of the studies, we found a relationship between adaptive behavior with the ADI-R [65], but not the ADOS-2 [66,67]. There are several explanations for the different correlation of the VABS-3 with the ADOS-2 and ADI-R [65]. The first is that the ADOS-2 reflects the current rate of symptoms, while the ADI-R maps a longer developmental period [68]. Namely, in the case of the ADI-R for children older than 5 years, several questions and evaluations of the social area and communication point to the period between 4–5 years, and some issues relating to stereotyped behavior concern an even younger period. So, individuals who have had more early social deficits according to the ADI-R also have more impaired adaptive behavior. Furthermore, some items in the ADI-R and VABS-3 are overlapped, which can contribute to a stronger association between the two instruments. Last, but not least, it is necessary to take into the account the fact that adaptive behavior and the rate of symptoms according to the ADI-R are assessed by parents, while in the ADOS-2 the child is evaluated by an expert, not taking into consideration the judgment of the parents.

The results of the current study have clinical implications regarding the interventions in children with ASD. The identification and treatment of sleep problems in ASD are imperative for improving sleep, as well as children’s daytime functioning and behavior. The first step should always be parent counseling to implement healthy sleep practices and behavioral interventions. The most common practices include sleep hygiene, graduated extinction, scheduled awakenings, bedtime fading, and also special behavioral training for the parents and children [4,69]. A non-pharmacological intervention is the preferred route, over pharmacologic therapy. Melatonin is one of the most frequently used and investigated substances in children with ASD [4]. Melatonin has been shown to be effective in treatment-reduced sleep onset insomnia and improved delayed-sleep phase syndrome. A meta-analysis of 18 studies on melatonin treatment in ASD has found significant improvements, with large effect sizes in sleep duration, and also improvements in sleep onset latency, and night-time awakenings, with minimum side effects [53]. In addition, a greater effectiveness of melatonin has also been observed in dual therapy, alongside a cognitive-behavioral intervention [69]. This corresponds with the results of a meta-synthesis showing that no single intervention is effective across all of the sleep problems in ASD children. Melatonin, behavioral interventions, and parental education/education program interventions seem to be the most effective at reducing sleep problems, compared with other interventions [70]. Mounting evidence indicates the potential effect of melatonin administration on other mental and physical health comorbid problems associated with ASD with a negative impact on sleep. It has been shown that melatonin has beneficial effects on anxiety, gastrointestinal dysfunction, or sensory processing. In this way, melatonin could at least partially exert positive effects on sleep in the subjects with ASD by its effects on these alternative routes [58]. Still, there is a need for additional research in order to provide clinicians with evidence-based facts for best practice [70]. Several limitations apply to this study. They include the small sample size, and due to this fact no additional factors were included in the analyses, such as gender or individual types of sleep disorders. In addition, no control group was included allowing for the comparison of the effects of sleep disturbances on adaptive behavior in neurotypical controls. The sleep measures were obtained by questionnaires filled in by parents, thus, this subjective method may be a source of bias. Since we determined the aMT6s levels in a single spot urine sample, possible shifts in circadian melatonin secretion might have been missed. Possible factors influencing the levels of melatonin, such as seasonality and environmental exposure to light, were also not considered.

## 5. Conclusions

In this study, we demonstrated that children with ASD with sleep problems are more likely to present more deficits in daily functioning, moreover, that sleep problems may serve as predictors of their adaptive functioning. The results show that in predicting the adaptive behavior based on sleep characteristics, there is need to also consider the age of a child. In our analyses, the preferred model was that for the Socialization domain using the age-adjusted Sleep score as a predictor. We have shown that the Sleep score, based on the Children’s Sleep Habits Questionnaire, correlated with the Externalizing maladaptive behavior, i.e., the presence of Sleep problems predicted the presentation of challenging behaviors, such as aggression, self-injury, or tantrums. The sleep problems also predicted the Internalizing maladaptive behavior, with only a negligible contribution of age. We found that the model for predicting Externalizing maladaptive behavior from the age-adjusted Sleep score even outperformed the model for predicting Internalizing maladaptive behavior in classification accuracy.

Validated questionnaires are commonly used tools for assessing sleep problems in children. They are subjective methods of assessment, and a potential source of bias. Abnormal nighttime melatonin secretion was implicated in playing a role in sleep problems. In our study, we investigated if the morning urinary levels of normalized 6-sulfatoxymelatonin, a waste product reflecting nighttime melatonin production, could serve as a predictor of adaptive functioning and sleep problems. Our results have not supported this hypothesis. In our sample, however, the urinary levels of normalized aMT6s predicted the severity of ASD core symptoms in the ADOS-2 domains, Social affect and the Calibrated Severity Score. This suggests that abnormal melatonin secretion may participate in the pathophysiology of ASD and its behavioral presentations, but by other mechanisms rather than the effect on sleep.

Our results emphasize the importance of assessing sleep problems as a modifiable predictor of behavior. Interventions focused on improving the sleep patterns may have important effects on daytime functioning, behavior, and the developmental outcomes of children with ASD.

## Figures and Tables

**Table 1:** Sample characteristics (n = 56; 50 boys, 6 girls).

	Mean	SD	Median	Minimum	Maximum
age (years)	5.3	2.4	4.8	2.8	13.3
aMT6s (ng/mL)	173.7	114.4	158.6	11.0	590.7
creatinine (mg/mL)	0.9	0.3	0.9	0.1	1.4
aMT6s/creatinine (ng/mg)	197.3	130.5	157.9	14.1	739.0
CSHQ Sleep score	44.5	6.3	45.0	33.0	58.0
**ADI** **-R**					
SUM_A (%)	0.4	0.2	0.4	0.1	0.9
SUM_B (%)	0.5	0.2	0.5	0.1	0.9
SUM_C (%)	0.3	0.2	0.3	0.1	0.8
**ADOS-2**					
SA	7.1	1.5	7.0	5.0	10.0
RRB	8.7	1.3	9.0	4.0	10.0
CSS	7.9	1.4	8.0	6.0	10.0
**Adaptive behavior**					
ABC	70.6	10.5	70.0	51.0	98.0
COM	67.0	18.2	67.0	24.0	113.0
DLS	76.4	10.3	76.0	54.0	98.0
SOC	68.3	14.9	66.0	38.0	98.0
IN	18.1	2.5	19.0	12.0	22.0
EX	18.5	2.4	18.0	11.0	22.0

aMT6s—6-sulfatoxymelatonin; CSHQ—Children’s Sleep Habits Questionnaire; SUM_A—Qualitative abnormalities in reciprocal social interaction (ADI-R); SUM_B—Qualitative abnormalities in communication (ADI-R); SUM_C—Restricted, repetitive patterns of behavior and interests (ADI-R); SA—Social affect (ADOS-2); RRB—Restricted and repetitive behavior (ADOS-2); CSS—Calibrated Severity Score (ADOS-2); ABC—Adaptive behavior composite score; COM—Communication; DLS—Daily living skills; SOC—Socialization; IN—Internalizing maladaptive behavior; EX—Externalizing maladaptive behavior.
